# Genetic and Phenotypic Characterization of in-Host Developed Azole-Resistant *Aspergillus flavus* Isolates

**DOI:** 10.3390/jof7030164

**Published:** 2021-02-25

**Authors:** Jochem B. Buil, Jos Houbraken, Monique H. Reijers, Jan Zoll, Maurizio Sanguinetti, Jacques F. Meis, Paul. E. Verweij, Willem J.G. Melchers

**Affiliations:** 1 Department of Medical Microbiology, Radboud University Medical Center, 6525 GA Nijmegen, The Netherlands; Jan.Zoll@radboudumc.nl (J.Z.); jacques.meis@gmail.com (J.F.M.); Paul.Verweij@radboudumc.nl (P.E.V.); Willem.Melchers@radboudumc.nl (W.J.G.M.); 2 Center of Expertise in Mycology Radboudumc/CWZ, 6525 GA Nijmegen, The Netherlands; Monique.Reijers@radboudumc.nl; 3 Westerdijk Fungal Biodiversity Institute, 3584 CT Utrecht, The Netherlands; j.houbraken@wi.knaw.nl; 4 Department of Pulmonology, Radboud University Medical Center, 6525 GA Nijmegen, The Netherlands; 5 Dipartimento di Scienze Biotecnologiche di Base, Cliniche Intensivologiche e Perioperatorie, Università Cattolica del Sacro Cuore, 00168 Roma, Italy; maurizio.sanguinetti@unicatt.it; 6 Dipartimento di Scienze di Laboratorio e Infettivologiche, Fondazione Policlinico Universitario A. Gemelli IRCCS, 00168 Roma, Italy; 7 Department of Medical Microbiology and Infectious Diseases, Canisius Wilhelmina Hospital (CWZ), 6532 SZ Nijmegen, The Netherlands

**Keywords:** *Cyp51A*, azole resistance, chronic pulmonary aspergillosis, aspergillosis, Y119F, acquired resistance

## Abstract

*Aspergillus flavus* is a pathogenic fungal species that can cause pulmonary aspergillosis, and triazole compounds are used for the treatment of these infections. Prolonged exposure to azoles may select for compensatory mutations in the *A. flavus* genome, resulting in azole resistance. Here, we characterize a series of 11 isogenic *A. flavus* strains isolated from a patient with pulmonary aspergillosis. Over a period of three months, the initially azole-susceptible strain developed itraconazole and voriconazole resistance. Short tandem repeat analysis and whole-genome sequencing revealed the high genetic relatedness of all isolates, indicating an infection with one single isolate. In contrast, the isolates were macroscopically highly diverse, suggesting an adaptation to the environment due to (epi)genetic changes. The whole-genome sequencing of susceptible and azole-resistant strains showed a number of mutations that might be associated with azole resistance. The majority of resistant strains contain a Y119F mutation in the *Cyp51A* gene, which corresponds to the Y121F mutation found in *A. fumigatus*. One azole-resistant strain demonstrated a divergent set of mutations, including a V99A mutation in a major facilitator superfamily (MSF) multidrug transporter (AFLA 083950).

## 1. Introduction

Chronic pulmonary aspergillosis (CPA) is a relatively uncommon pulmonary disease, occurring in apparently nonimmunocompromised patients. The disease often affects patients with other respiratory disorders such as chronic obstructive pulmonary disease, tuberculosis, nontuberculous mycobacteria (NTM) infection, prior pneumothorax or treated lung cancers [1,2]. *Aspergillus fumigatus* is the causative species of CPA in the majority of patients, but other species such as *A. niger*, *A. terreus* and *A. flavus* may also be cultured from patients with CPA [3]. Subacute invasive aspergillosis (SAIA) is another clinical presentation of pulmonary aspergillosis occurring in mild immunocompromised patients. The clinical and radiological features are very similar to CPA, but the progression of the disease is more rapid. In contrast to CPA, serum galactomannan is often positive in SAIA. However, SAIA has a slower course of progression (one to three months) compared to acute invasive aspergillosis [1]. Symptomatic or progressive CPA is treated primarily with itraconazole, but voriconazole and posaconazole are potential alternative agents [1,4]. Patients with SAIA should always be treated with antifungals according to the CPA guideline. Voriconazole and isavuconazole are regarded as first-line agents for these patients [1,4].

Azole resistance in *A. fumigatus* is reported in many countries worldwide and challenges the treatment of *Aspergillus* diseases [5,6]. Although azole resistance has also been described in *A. flavus* isolates, the mechanism of azole resistance has been less extensively studied in *A. flavus* compared to *A. fumigatus.* Azole-resistant infections may be due to the direct inhalation of resistant spores present in the environment. Alternatively, azole resistance may develop during azole treatment, characterized by a phenotype switch from drug susceptible to drug resistant [7,8]. The latter occurs primarily in patients with chronic forms of aspergillosis receiving long-term therapy and especially in those with pulmonary cavities [9,10].

Here, we describe a patient who was treated for SAIA. During antifungal treatment, several *A. flavus* isolates were cultured. The sequentially cultured isogenic isolates in this study showed a highly variable macroscopic morphology. The first two isolates appeared to be azole susceptible, and the following nine isolates had increased MICs for voriconazole and itraconazole. Azole resistance generally comes with a fitness cost, which may become apparent through growth variation when resistant isolates are cultured in the absence of azole pressure under laboratory conditions [11]. In *A. fumigatus*, similar variations in colony morphology were observed in sequential cultures [12]. To further understand in-host adaptation dynamics of *A. flavus* during antifungal treatment, we performed genetic analysis of 11 cultured *A. flavus* isolates from consecutive cultures obtained from a single patient during azole treatment for SAIA.

## 2. Materials and Methods

### 2.1. Origin of Strains and Antifungal Treatment 

**Figure 1:**
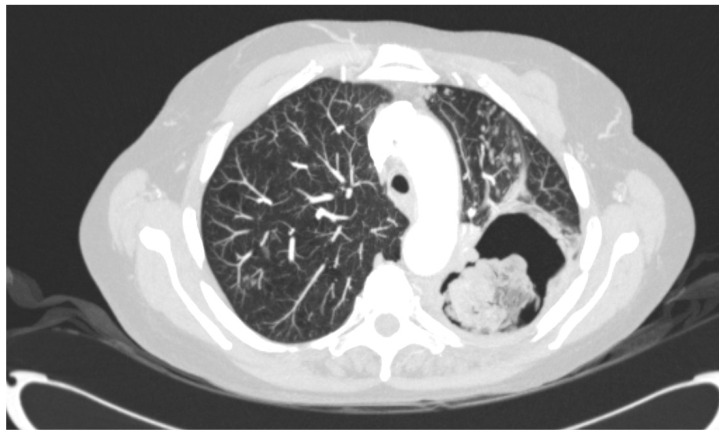
Chest computed tomography image showing a large pulmonary cavity in the left upper lobe with a mass lying in the cavity.

**Figure 2:**
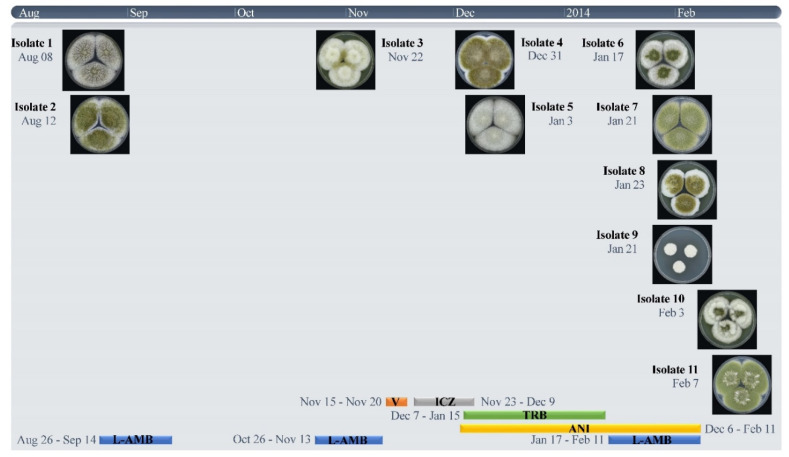
Timeline of the antifungal treatment and *A. flavus* culture morphology. VCZ, voriconazole; ICZ, itraconazole; L-AMB, liposomal amphotericin B; TRB, terbinafine; ANI, anidulafungin. The dates that clinical samples were obtained from the patient are indicated in the Figure, and the numbering of the isolates is based on the date the cultures became positive. Isolates 7 and 9 were cultured from the same clinical material but showed different morphologies. Isolate 8 was cultured from a clinical sample obtained 2 days after Isolates 7 and 9. This culture was earlier positive than the cultures of Isolate 9.2.2. Strains, phenotypic analysis, antifungal susceptibility and typing.

The 11 strains used in this study were cultured from a 66-year-old female with COPD Gold IIA. One and a half years prior to the first *A. flavus* culture, computed tomography (CT) of the lungs showed a cavity, and subsequent biopsy culture showed *Mycobacterium intracellulare*, which was treated with clarithromycin. Eight months later, CT showed an aspergilloma in the pre-existing cavity, and the patient was treated with voriconazole for six weeks. As the patient’s condition worsened, with progressive weight loss and recurrent hemoptysis, she was referred to the University Medical Centre (Nijmegen, the Netherlands) for the optimization of nontuberculosis mycobacterial treatment. Her antimycobacterial therapy was changed to clarithromycin, rifampicin and ethambutol. No clinical improvement was observed, and two sputum cultures showed *A. flavus* (Isolates 1 and 2), which prompted a diagnostic work-up for CPA (Figure 1 and Figure 2). The serum galactomannan (GM) index (2.0), *Aspergillus* IgG (43 mg/L) (ImmunoCap) and IgE antibodies (0.44 kU/L) were positive, which, together with her CT scan abnormalities, were consistent with SAIA. Antifungal treatment was again started with liposomal amphotericin B (L-AmB). Although *A. flavus* may be less susceptible to L-AmB treatment, the concurrent use of rifampicin precluded azole therapy, and L-AmB was chosen as the second-best option. After six weeks, both L-AmB and rifampicin were stopped due to side effects. The serum GM index had declined to 0.6. The patient was subsequently treated with voriconazole, which was changed to itraconazole after two weeks due to visual disturbances. During this therapy, *A. flavus* was cultured from sputum, which now showed an azole-resistant phenotype (Isolate 3)*,* and antifungal treatment was changed to anidulafungin. In addition, terbinafine was started as the terbinafine MICs were low, and the combination was aimed to prevent or delay further resistance selection. As symptoms worsened and serum GM increased to 3, L-AmB was restarted after five weeks. As no improvement was observed despite continued antifungal and tuberculostatic agents, antifungal treatment was stopped, and the patient was discharged. The patient remained stable for some time after discharge but eventually died after 12 months.

Over a six-month period, 11 isolates were cultured from the patient, and all were morphologically identified as *A. flavus* at the Centre of Expertise in Mycology Radboudumc/CWZ. Isolates were stored at −80 ℃ in 10% glycerol. In vitro susceptibility testing of the isolates was performed according to the EUCAST broth microdilution reference method for molds (E.def 9.2) [13]. Isolates were tested at a final drug concentration range of 0.032–16 mg/L for itraconazole, voriconazole and posaconazole. The endpoint defined as no growth was visually determined. Susceptibility testing was performed at the time the isolates were cultured, and the results were confirmed by reviving the isolates and repeating the susceptibility testing. Short tandem repeat (STR) typing was performed as described previously using three sets of three markers (AflaSTR2, AflaSTR3 and AflaSTR4) [14,15]. The isolates were phenotyped by studying the colony morphology on agar media (creatine agar (CREA), Czapek yeast extract agar (CYA) incubated at 25, 37 and 40 °C, CYA supplemented with 5% NaCl (CYAS), dichloran 18% glycerol agar (DG18), malt extract agar (MEA), Sabouraud dextrose agar (SAB) and yeast extract sucrose (YES) agar). The agar media were inoculated in a three-point position and incubated for 7 days at 25 °C (unless stated otherwise) in darkness. The composition of the media is according to Samson et al. [16]. Partial β-tubulin (*BenA*), calmodulin (*CaM*) and RNA polymerase II second largest subunit (*RPB2*) sequencing were performed as previously described [17].

### 2.2. Whole-Genome Sequencing and Analysis

Genomic DNA was extracted from conidia. Conidia were suspended in Tris–EDTA buffer (pH 8, supplemented with 1% SDS, 2% Triton × 100 and 100 mM NaCl). The suspension was shaken for 30 min at 70 °C. DNA was extracted using phenol/chloroform and purified using the QIAamp DNA Blood Mini kit (Qiagen, Aarhus, Denmark). A fragmented genomic DNA library was prepared using a Nextera XT DNA sample preparation kit (Illumina, San Diego, USA). Subsequent sequencing was conducted in a paired-end 2 × 150 bp mode using an Illumina NextSeq500 machine. Reads were mapped to the *Aspergillus flavus* NRRL3357 (Assembly GCA_000006275.2, EnsemblFungi) and *Aspergillus oryzae* RIB40 (ASM18445v3) (Assembly GCA_000184455.3, EnsemblFungi) reference genomes using CLC Genomics Workbench 12 (Qiagen, Aarhus, Denmark). Single-nucleotide polymorphism (SNP) detection and variant comparisons were conducted using CLC Genomics Workbench 12 (Qiagen) and the Basic Variant Detection method with ploidy 1, a minimum coverage of 5 and a minimum probability of 0.8.

**Figure 3:**
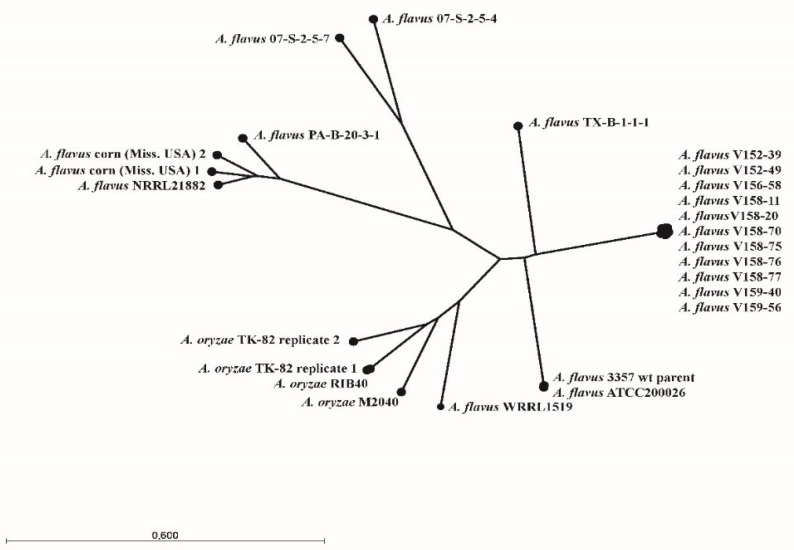
Neighbor-joining phylogenetic relationships of sequential isolated *A. flavus* strains and 14 *A. flavus* and *A. oryzae* strains obtained from the NCBI SRA database based on whole-genome sequencing data. The phylogenetic relationship was calculated from a distance matrix based on the average nucleotide identity.

Whole-genome sequencing data from 14 *A, flavus* and *A. oryzae* strains were obtained from the NCBI SRA database (Appendix A Table A1). Contigs were generated by de novo assembly using sequencing reads from all clinical samples and reference strains. Whole-genome alignment was performed on sets of contigs with a minimum size of 10 kb. Whole-genome alignment and pairwise comparison were performed using the Whole-Genome Alignment tool implemented in CLC Genomic Workbench version 20.0.4 with default settings [18]. The generated distance matrix, based on the average nucleotide identity (ANI), was used to calculate the neighbor-joining phylogenetic relationships (Figure 3).

SNP-calling on the various clinical samples was done by mapping reads of individual strains to contigs obtained from strain V159-40 as a reference genome. Variants were determined by the Basic Variant Detection tool in CLC Genomics Workbench version 20. The ploidy number was set to one and the minimum frequency filter to 95%. Default values were used for all other parameters and variant filters. Sequences containing SNPs present in a total of 177 positions were aligned using MAFFT [19]. A maximum-likelihood phylogenetic tree was calculated using CLC Genomics Workbench version 20.

## 3. Results

### 3.1. Strains, Phenotypical Analysis and Genotyping

Partial *BenA*, *CaM* and *RPB2* sequencing confirmed the morphological species identification as *A. flavus*. All isolates had identical *BenA*, *CaM* and *RPB2* sequences. The results of the susceptibility testing are shown in Table 1. Isolates 1 and 2 had low MICs for the azoles, and the azole MICs of Isolates 3–11 were increased compared to Isolates 1 and 2. Repeated testing of isolates showed similar MICs (within two dilution steps) for all isolates, except Isolate 4. For Isolate 4, the increased MICs could not be reproduced, even after subculturing the isolate on azole-containing agar first. The MIC of amphotericin B was either 1 or 2 mg/L for all isolates, and the MIC of anidulafungin ranged from 0.016 to 0.025 mg/L (Table 1).

**Figure 4:**
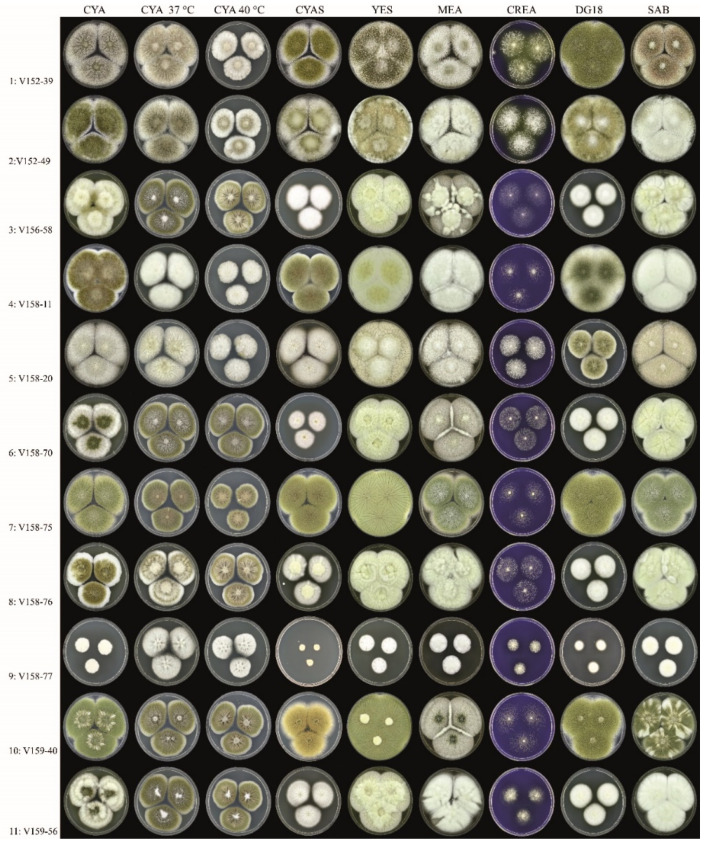
Overview of the phenotypic diversity of *A. flavus* isolates (all obverse, 7 days at 25 °C, unless stated otherwise). Columns, left to right: Czapek yeast extract agar (CYA), CYA incubated 37 °C, CYA incubated 40 °C, CYA supplemented with 5% NaCl (CYAS), yeast extract sucrose (YES), malt extract agar (MEA), creatine agar (CREA), dichloran 18% glycerol agar (DG18), Sabouraud dextrose agar (SAB). Rows, top to bottom: Isolates 1–11.

**Figure A1:**
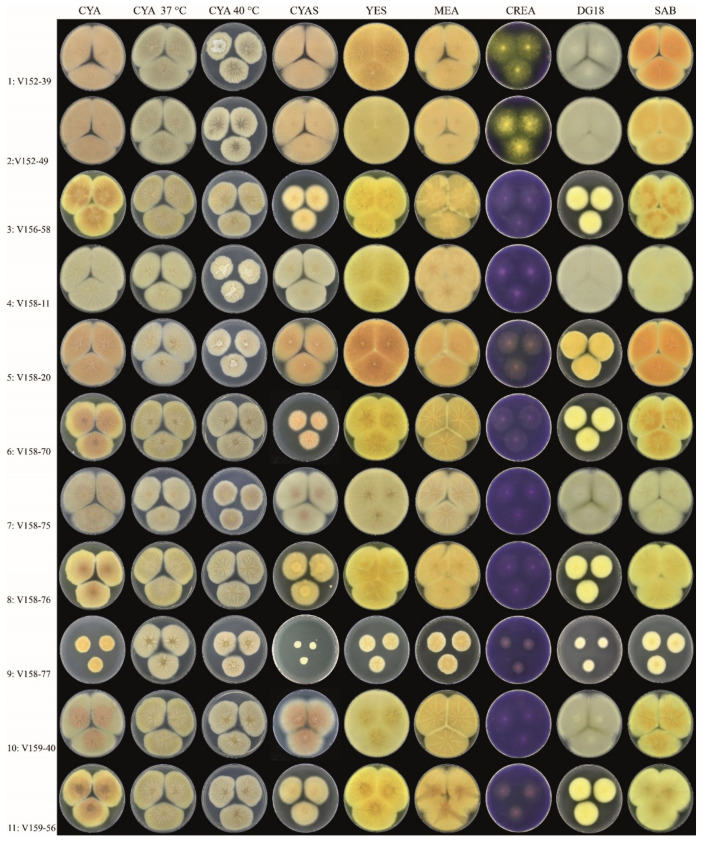
Overview of the phenotypic diversity of *A. flavus* isolates (all reverse, 7 days at 25 °C, unless stated otherwise). Columns, left to right: CYA, CYA incubated 37 °C, CYA incubated 40 °C, CYAS, YES, MEA, CREA, DG18, SAB. Rows, top to bottom: Isolates 1–11.

Growth analysis of the isolates at eight different conditions revealed a diverse colony morphology (Figure 4 and Figure A1), and none of the isolates were phenotypically identical. Isolates 1 and 2 showed typical spreading *A. flavus* colonies, with abundant sclerotia production on CYA incubated at 25 °C and yellow-green-colored conidia. Isolate 5 resembled Isolates 1 and 2, though sporulation was absent on most agar media, and growth was reduced on DG18. Isolates 3, 6, 8 and 11 had similar colony diameters, and of those isolates, 6 and 11 had a similar degree of sporulation. Compared to Isolates 1 and 2, these isolates had smaller colony diameters on CYAS and DG18. Isolate 4 was unique in producing greenish-brown-colored conidia, and Isolate 9 had very restricted growth, unlike all other isolates. Isolates 7 and 10 resembled each other in colony diameters, though Isolate 7 sporulated more strongly. Short tandem repeat genotyping revealed full genetic relatedness between all isolates, suggesting that the isolates originate from the same parent strain. The tandem repeat numbers are shown in Table 1.

### 3.2. Whole-Genome Sequence Analysis

**Figure A2:**
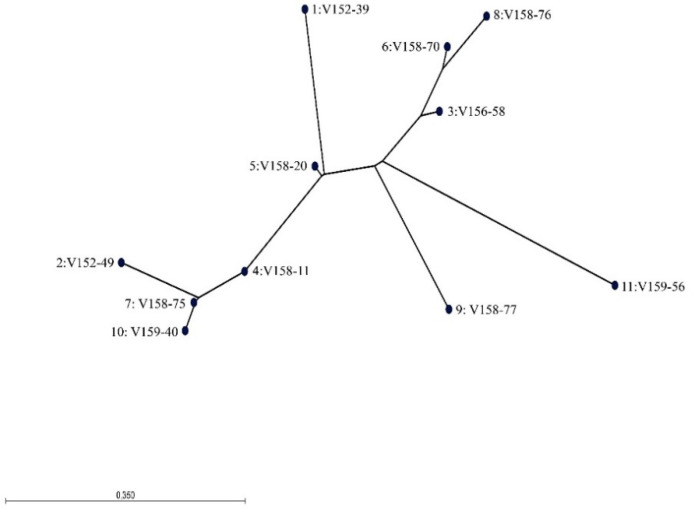
Maximum-likelihood phylogenetic tree of the 11 *A. flavus* isolates.

*Aspergillus flavus* NRRL3357 and *A. oryzae* RIB40 were used as reference genomes for read mapping based on an assessment of mapping quality and coverage statistics; a mean coverage of approximately 40 and a mean mapping quality score of 40 were found across the sequenced isolates. A total of 31 nonsynonymous SNPs, absent in early Isolates 1 and 2, were identified in later isolates (Table 2). The *cyp51A* mutation Y119F (corresponding to the Y121F mutation in *A. fumigatus*) was found in Isolates 3 and 5-11 but not in Isolate 4. Isolates 3, 5, 6, 8, 9 and 11 had a mutation in a gene with putative C-4 methyl sterol oxidase function, resulting in a stop codon at Locus 35. Isolate 4 did have a point mutation in a putative major facilitator superfamily (MFS) transporter (AFLA_083950). Phylogenetic relationship analysis based on whole-genome sequencing data confirmed the results based on short tandem repeat genotyping. All sequential cultured strains showed a genetic relationship (Figure 3). Phylogenetic relationship analyses of the 11 cultured *A. flavus* strain showed that Isolates 2, 7 and 10 were genetically highly similar to each other. Isolates 3, 6, 8, 9 and 11 were more genetically related to each other (Figure A2).

## 4. Discussion

Here, we describe the characteristics of 11 *A. flavus* isolates that were cultured during the treatment of SAIA in a patient with chronic lung disease. All isolates were identified as *A. flavus* and had identical partial *BenA*, *CaM* and *RPB2* gene sequences, showing high genetic similarity. STR analysis and whole-genome sequencing further confirmed the isogeneity of all isolates. The isolates were macroscopically very diverse and later isolates showed decreased in vitro susceptibility to azoles.

Azole resistance is an increasing problem worldwide, especially in *A. fumigatus.* [20]. However, reports on azole resistance in *A. flavus* are sparse, and the resistance mechanisms are generally unknown. Several single-nucleotide polymorphisms (SNPs) have been reported in the *A. flavus Cyp51* genes encoding lanosterol 14 alpha-demethylase. This protein is the target of azoles, and the *A. flavus* genome contains three orthologs of this gene. One study demonstrated S196F, A324P, N423D and V465M polymorphisms in the *Cyp51C* gene in an azole-resistant isolate [21]. However, another study identified the same SNPs in both azole-susceptible and azole-resistant isolates, arguing against the association of these mutations for azole resistance [22]. Another study suggested the role of the S240A SNP in the *Cyp51C* gene. They found an S240A SNP in an isolate with increased voriconazole MICs and confirmed its role in voriconazole tolerance by transformations [23]. However, again, this SNP was also found in many azole-susceptible isolates [21,22,24]. The last SNP in *Cyp51C* that was suggested to play a role in azole susceptibility is Y319H [24]. In laboratory-selected resistant isolates using voriconazole stress, both *Cyp51A* and *Cyp51B* mutations were identified compared to the parental strain. K197N, Y132N+T469S and K197N+D282E+M288L SNPs were found in the *Cyp51A* of isolates with increased voriconazole MICs, and H399P+D411N and T454P+T486P were observed in the *Cyp51B* of 2 other isolates [25]. However, a direct relationship with azole resistance was not established. In addition, other non-*cyp51-*mediated mechanisms, such as azole efflux, due to the overexpression of ATP-binding cassettes (ABC) and MFS transporters, have been suggested to play a role in azole resistance in *A. flavus*. [26].

We identified a novel Y119F substitution in the *cyp51A* gene, which seemed to be the cause of the azole resistance phenotype in eight of nine azole-resistant isolates. Although the relation of the Y119F mutations and azole resistance is not formally proven, strong indirect evidence is available. First, the mutation was observed in azole-resistant strains, and the genetically identical parental isolates without this mutation were susceptible. Secondly, in *A. fumigatus,* the homolog Y121F mutation is directly linked to increased voriconazole MICs, and this phenotype is supported by a CYP51A homology protein model [27,28]. Furthermore, Y119F-corresponding homologous mutations in other fungal species have similar effects on azole susceptibility. For example, the homolog Y132F mutation has been described in azole-resistant *Candida albicans* and was also found in azole-resistant *C. auris* and *C. parapsilosis* isolates [29,30,31]. The homolog Y145F mutation in *C. neoformans* [32] and the Y136F mutation in the *Histoplasma capsulatum cyp51A* gene [33] were associated with reduced voriconazole susceptibility. Furthermore, the homolog Y137F mutation in *Mycosphaerella graminicola* [34] and the Y136F mutation in *Uncinula necator* [35] are associated with resistance to triadimenol, a triazole that shows structurally more resemblance to voriconazole than to the long-tailed triazole itraconazole and posaconazole. Interestingly, the *C. neoformans* and *H. capsulatum* strains with Y145F and Y136F mutations remained susceptible to itraconazole and posaconazole [32,33]. Thus, the increased MICs of voriconazole observed in our isolates are likely explained by the Y119F mutation in the *Cyp51A* gene. However, itraconazole showed paradoxical growth (eagle effect) in our clinical isolates, where significant growth inhibition was seen at relatively low concentration but only minimal growth inhibition at higher concentrations of itraconazole. Whether the Y119F substitution is causing this phenomenon and whether itraconazole remains clinical effective is not clarified. 

Another mutation resulting in a stop codon at Locus 35 found in six of nine azole-resistant isolates might also be involved in adaptation to azole stress. Orthologs of the AFLA_115530 gene, which has a putative C-4 methyl sterol oxidase function, are involved in sterol biosynthesis. The orthologs ERG25 and ERG25b in *A. fumigatus* are upregulated during azole stress [36]. Furthermore, in vitro itraconazole selection was pressure-selected for a mutation in the ERG25 gene of *A. fumigatus*. [37]. Although the precise function and its effect on azole susceptibility are largely unknown, azole stress may have resulted in the selection of this mutation. In addition to azole treatment, the patient was treated with L-AmB and anidulafungin, which may select for mutations in the ERG genes or FKS gene. However, none of the isolates had increased L-AmB MICs and SNPs in the FKS gene were not identified.

Isolate 4 did not harbor the Y119F *Cyp51A* mutation or the mutation putative C-4 methyl sterol oxidase. Initial testing showed increased MICs for itraconazole and voriconazole, but after reviving the stored isolate, repeated testing could not confirm the initially observed phenotype. It could be that this isolate harbored a nonstable resistance mechanism that was lost during storage and subculturing. However, growing the isolate under azole pressure did not reproduce the increased MICs (data not shown). Alternatively, the stored isolate might not have been a pure culture, and reviving and subculturing may have resulted in the selection of a faster-growing nonresistant isolate. Whole-genome sequencing revealed a mutation in the putative MFS transporter (AFLA_083950) gene of this isolate. In *A. fumigatus,* azole stress increases the expression of the ortholog AFU1G15490 gene, and thus this gene may be involved in the development of azole tolerance [38]. However, further studies would be required to confirm this observation.

Isolates 3, 5, 6, 8 and 11 share a similar mutation pattern and also have similar growth rates (Table A2). Compared to isolates 1 and 2 (wild-type phenotype), all strains grow slower on DG18 and, with the exception of Isolate 5, slower on CYAS (Figure 4). These two agar media have a lowered water activity due to the presence of glycerol (DG18) and NaCl (CYAS). It can be speculated that the evolution of the isolate in the patient led to reduced stress resistance against lowered water activity, a condition not present in the human lung. In contrast, *A. fumigatus* isolates obtained from humans with suspected invasive pulmonary aspergillosis and dogs with sino-orbital aspergillosis have a higher growth rate on DG18 compared with environmental strains [39]. Isolate 4 has four mutations (R52G, V99A, A561T, T159I) that are not observed in the other isolates. The production of greenish-brown-colored conidia is a unique macro-morphological feature of this isolate and might be linked to one or a combination of these mutations. The function of two genes with mutations is unknown; the other two are an MSF transporter and a global gene regulator VeA. Isolates 7 and 10 resemble each other and these two strains only have the Y119F mutation. These strains have similar growth rates as Isolates 1 and 2 but lack the production of sclerotia and acid production on CREA.

Phylogenetic analysis of the isolates showed that Isolates 2, 7 and 10 were highly similar, indicating these isolates were from the same subpopulation. Isolate 2 was isolated at the beginning of the infections, whereas Isolate 10 was cultured six months later. However, several less-related *A. flavus* isolates were cultured between Isolates 2 and 10, which possibly represent different subpopulations in the lungs of the patient. This indicates that the patient was initially infected with a single *A. flavus* strain, which resulted in several subpopulations. Furthermore, it also indicates that these subpopulations of *A. flavus* were present in the lungs of the patient at the same time. The first azole-resistant isolates were cultured already after one week of voriconazole treatment. It could be that the resistant isolates acquired the resistance-associated mutations during the earlier six weeks’ voriconazole treatment and thus were already present in the lung of the patient. It could be that these resistant isolates were selected during the second treatment with voriconazole. This would explain how the resistant strains could be isolated after a very short course of voriconazole treatment. The clinical samples did not grow strains from every population in every culture that was taken from this patient. Thus, even when a culture from a patient with aspergillosis is positive, it could be that another isogenic population of *Aspergillus* is present in the lung with a different susceptibility phenotype if SNPs conferring resistance were selected in this subpopulation. Accordingly, as not all subpopulations will be detected in a single culture, patients with CPA treated with antifungal agents should be cultured repeatedly, especially if a pulmonary cavity is present in the lungs or the patient does not improve clinically.

The sequentially cultured isolates in this study were morphologically very diverse. Interestingly, STR and genomic analysis showed that all isolates were isogenic, indicating that the patient was infected with one single isolate. Only few SNPs were found in later isolates compared to the first two cultured isolates. The variable colony morphology may be due to fitness costs associated with resistance development, nutrient availability or adaptation to other host stressors [9,40]. In vitro studies indicate that azole stress may result in morphological variation. The in vitro selection of azole resistance using posaconazole resulted in apparent morphological changes in *A. fumigatus* colonies [41]. Similar morphological changes were observed in azole-resistant isolates exposed to itraconazole selection pressure [11]. Morphological variation was also seen in consecutive *A. fumigatus* isolates cultured from a patient with recurrent invasive aspergillosis extensively treated with several antifungals [12]. It is possible that the observed mutations in our isolates underlie the variable phenotypes, but other underlying (epi)genetic mechanisms may be undiscovered. The precise role of the mutations observed in our study regarding the morphological variation remains unknown and could not be explained by the function of the mutated genes alone. Further transformation studies would be needed to further elaborate on the role of these genes. Our observations show that genetically related isolates can be very different morphologically, and genetic relatedness cannot be excluded based on morphology alone [42]. 

## Figures and Tables

**Table 1:** Overview susceptibility and short tandem repeat (STR) typing of 11 sequentially cultured *A. flavus* isolates MIC minimal inhibitory concentration.

Strain Number	Isolation Date		MIC (mg/L)				STR Numbers		
		ITC	VOR	POSA	AMB	ANI ^c^	2	3	4
1 V152-39	8-8-2013	0.5	1	0.5	1	0.063	23-14-17	8-22-12	5-7-2011
2 V152-49	12-8-2013	1	4	0.25	2	0.031	23-14-17	8-22-12	5-7-2011
3 V156-58	22-11-2013	16 ^a^	>16	0.5	2	0.031	23-14-17	8-22-12	5-7-2011
4 V158-11	31-12-2013	>16 ^a,b^	>16 ^b^	1	2	0.063	23-14-17	8-22-12	5-7-2011
5 V158-20	3-1-2014	16 ^a^	16	0.5	1	0.016	23-14-17	8-22-12	5-7-2011
6 V158-70	17-1-2014	2	>16	0.5	2	0.016	23-14-17	8-22-12	5-7-2011
7 V158-75	21-1-2014	>16 ^a^	>16	1	2	0.125	23-14-17	8-22-12	5-7-2011
8 V158-76	23-1-2014	>16 ^a^	>16	1	2	0.125	23-14-17	8-22-12	5-7-2011
9 V158-77	21-1-2014	1	16	0.25	2	0.25	23-14-17	8-22-12	5-7-2011
10 V159-40	3-2-2014	>16 ^a^	>16	2	2	0.031	23-14-17	8-22-12	5-7-2011
11 V159-56	7-2-2014	1	>16	0.5	2	0.016	24-14-17	8-22-12	5-7-2011

STR, short tandem repeats; ITC, itraconazole; VOR, voriconazole; POSA, posaconazole; AMB, amphotericin B; ANI, anidulafungin. ^a^ Isolates showed paradoxical growth (eagle effect). Significant growth inhibition was seen at a relatively low concentration but only minimal growth inhibition at higher concentrations of itraconazole. ^b^ The MIC results could not be reproduced. Repeated testing showed a MIC of 0.25 mg/L for itraconazole and a MIC of 2 mg/L for voriconazole. ^c^ Repeating the MIC of anidulafungin resulted in a MIC of 0.016–0.032 mg/L for all isolates.

**Table 2:** Nonsynonymous mutations in azole-resistant *A. flavus* isolates compared to two isogenic azole-susceptible isolates.

			Amino Acid Change											
Chr.-Pos. ^a^	Allele		Isolate ^b^									*A. oryzae* Gene ^c^ (RefSeq Accession Numbers)	*A. flavus* Gene ^d^	Function
			3	4	5	6	7	8	9	10	11			
1-498965	T	A	I997N		I997N	I997N		I997N			I997N	AO090009000185 (XM_001816630)	AFLA_052710	GTPase activating protein
1-561401	A	G		R52G								AO090009000208 (XM_023234211)	AFLA_052490	unknown
1-1099800	C	T	A221T		A221T	A221T		A221T				AO090009000417 (XM_001816830)	AFLA_050600	Aldehyde dehydrogenase
1-3360498	T	C		V99A								AO090005001154 (XM_023232984)	AFLA_083950	MSF transporter
1-3370761	T	A	H25Q		H25Q	H25Q		H25Q	H25Q		H25Q	AO090005001151 (XM_023232999)	AFLA_083930	DnaJ domain protein
2-578295	C	T	P38L		P38L	P38L		P38L			P38L	AO090001000237 (XM_001818728)		Global gene regulator VeA
2-579927	G	A		A561T								AO090001000237 (XM_001818728)		Global gene regulator VeA
2-2642838	T	A	Y119F		Y119F	Y119F	Y119F	Y119F	Y119F	Y119F	Y119F	AO090003000205 (XM_001819367)	AFLA_036130	14-alpha sterol demethylase
2-3253116	T	C	K1757R		K1757R	K1757R		K1757R	K1757R		K1757R	AO090003000437 (XM_001819566)	AFLA_033810	NPC protein An-Mlp1
3-851374	A	G	I570T		I570T	I570T		I570T	I570T		I570T	AO090023000332 (XM_023235544)	AFLA_107440	Sla1
4-1887254	C	T		T159I								AO090012000739 (XM_023236608)		unknown
4-4726184	C	T	R669Q			R669Q		R669Q	R669Q		R669Q	AO090166000062 (XM_023237067)		unknown
7-2588623	C	A	E35*		E35*	E35*		E35*	E35*		E35*	AO090206000001 (XM_023233185)	AFLA_115530	C-4 methylsterol oxidase
8-891870	G	A	P445L									AO090103000145 (XM_023233427)	AFLA_010830	unknown

Mutations are described based on mapping to the *A. oryzae* RIB40 reference genome. *A. flavus* equivalent genes are indicated. ^a^ Mutations are indicated by the chromosome number and nucleotide position in the *A. oryzae* RIB40 reference genome. ^b^ Subsequent isolates are indicated by their numbers: 3: V156-58, 4: V158-11, 5: V158-20, 6: V158-70, 7: V158-75, 8: V158-76, 9: V158-77, 10: 159-40, and 11: 159-56. ^c^ Annotated gene names based on the *A. oryzae* RIB40 reference genome. ^d^ Annotated gene names based on from the *A. flavus* NRRL3357 reference genome. If no annotation was available, the row was left empty.

## Data Availability

The datasets presented in this study can be found in online repositories. The names of the repository/repositories and accession number(s) can be found in the article.

## Appendix A

**Table A1:** Strain information including accession numbers of the strains used for phylogenetic analysis based on whole-genome sequencing data.

	Accession Number	Organism	Strain	Origin	
1	SRR4142427	*A. flavus*	3357 wt parent	Unknown	USA
2	SRR5906257	*A. flavus*	WRRL1519	Almond nut	Cal. USA
3	SRR6914138	*A. flavus*	ATCC200026	Peanut	USA
4	SRR7615260	*A. flavus*	NRRL21882	Peanut	USA
5	SRR8554744	*A. flavus*	from corn (Miss. USA)	Corn	Miss. USA
6	SRR8556699	*A. flavus*	from corn (Miss. USA)	Corn	Miss. USA
7	SRR11596600	*A. flavus*	07-S-2-5-7	Corn	Louis. USA
8	SRR11596612	*A. flavus*	07-S-2-5-4	Corn	Louis. USA
9	SRR12001143	*A. flavus*	TX-B-1-1-1	Soil	USA
10	SRR12001155	*A. flavus*	PA-B-20-3-1	Soil	USA
12	SRR7615261	*A. oryzae*	M2040	Industrial strain	South Korea
13	SRR1835311	*A. oryzae*	RIB40	Industrial strain	unknown
15	DRR163532	*A. oryzae*	TK-82 replicate 2	Industrial strain	unknown
16	DRR163631	*A. oryzae*	TK-82 replicate 1	Industrial strain	unknown
17	SAMN14278081	*A. flavus*	V152-39	Clinical sample	The Netherlands
18	SAMN14278082	*A. flavus*	V152-49	Clinical sample	The Netherlands
19	SAMN14278083	*A. flavus*	V156-58	Clinical sample	The Netherlands
20	SAMN14278084	*A. flavus*	V158-11	Clinical sample	The Netherlands
21	SAMN14278085	*A. flavus*	V158-20	Clinical sample	The Netherlands
22	SAMN14278086	*A. flavus*	V158-70	Clinical sample	The Netherlands
23	SAMN14278087	*A. flavus*	V158-75	Clinical sample	The Netherlands
24	SAMN14278088	*A. flavus*	V158-76	Clinical sample	The Netherlands
25	SAMN14278089	*A. flavus*	V158-77	Clinical sample	The Netherlands
26	SAMN14278090	*A. flavus*	V159-40	Clinical sample	The Netherlands
27	SAMN14278091	*A. flavus*	V159-56	Clinical sample	The Netherlands

**Table A2:** Overview of the amino acid changes of nine *A. flavus* isolates and the unique macromorphological characteristics.

Isolate	Amino Acid Change														Colony Diameter (mm, after 7 d, 25 °C)		Unique Macromorphological Characters Compared to Isolates 1–2 ^a^
	I997N	R52G	A221T	V99A	H25Q	P38L	A561T	Y119F	K1757R	I570T	T159I	R669Q	E35*	P445L	CYAS	DG18	
3	x		x		x	x		x	x	x		x	x	x	36–38	32–34	Colony diameters smaller on DG18 and CYAS, sporulation variable
4		x		x			x				x				50–53	>60	Colony diameters slightly smaller on CYAS; greenish-brown-colored conidia
5	x		x		x	x		x	x	x			x		54–58	36–38	Colony diameters smaller on DG18, slightly smaller on CYAS; sporulation generally absent or weak
6	x		x		x	x		x	x	x		x	x		26–30	32–34	Colony diameters smaller on DG18 and CYAS; sporulation variable
7								x							>60	>60	Colony diameters similar as Isolates 1–2; abundant sporulation on all agar media, except creatine agar
8	x		x		x	x		x	x	x		x	x		36–38	29–32	Colony diameters smaller on DG18 and CYAS; sporulation variable
9					x			x	x	x		x	x		8–10	16–18	Colony diameters restricted on all agar media; sporulation absent
10								x							54–58	>60	Growth diameters slightly smaller on CYAS, sporulation generally moderate or good
11	x				x	x		x	x	x		x	x		35–38	30–33	Colony diameters smaller on DG18 and CYAS; sporulation variable

^a^ Isolates 3–11 are compared to isolates 1 and 2; the latter two isolates present a typical *A. flavus* phenotype with a high growth rate on most media (CYAS: >60 mm, DG18: >60 mm), production of yellow-green-colored conidia and black sclerotia. Isolates 3–11 do not produce sclerotia or acid on creatine agar.
